# SARS-CoV-2 antibodies protect against reinfection for at least 6 months in a multicentre seroepidemiological workplace cohort

**DOI:** 10.1371/journal.pbio.3001531

**Published:** 2022-02-10

**Authors:** Emilie Finch, Rachel Lowe, Stephanie Fischinger, Michael de St Aubin, Sameed M. Siddiqui, Diana Dayal, Michael A. Loesche, Justin Rhee, Samuel Beger, Yiyuan Hu, Matthew J. Gluck, Benjamin Mormann, Mohammad A. Hasdianda, Elon R. Musk, Galit Alter, Anil S. Menon, Eric J. Nilles, Adam J. Kucharski, on behalf of the CMMID COVID-19 working group and the SpaceX COVID-19 Cohort Collaborative

**Affiliations:** 1 Centre for Mathematical Modelling of Infectious Diseases, London School of Hygiene & Tropical Medicine, London, United Kingdom; 2 Barcelona Supercomputing Center (BSC), Barcelona, Spain; 3 Catalan Institution for Research and Advanced Studies (ICREA), Barcelona, Spain; 4 Ragon Institute of MGH, MIT and Harvard, Cambridge, Massachusetts, United States of America; 5 Institut für HIV Forschung, Universität Duisburg-Essen, Duisburg, Germany; 6 Harvard Humanitarian Initiative, Cambridge, Massachusetts, United States of America; 7 Computational and Systems Biology Program, Massachusetts Institute of Technology, Cambridge, Massachusetts, United States of America; 8 Broad Institute of MIT and Harvard, Cambridge, Massachusetts, United States of America; 9 Space Exploration Technologies Corp, Hawthorne, California, United States of America; 10 Brigham and Women’s Hospital, Boston, Massachusetts, United States of America; University of Chicago, UNITED STATES

## Abstract

Identifying the potential for SARS-CoV-2 reinfection is crucial for understanding possible long-term epidemic dynamics. We analysed longitudinal PCR and serological testing data from a prospective cohort of 4,411 United States employees in 4 states between April 2020 and February 2021. We conducted a multivariable logistic regression investigating the association between baseline serological status and subsequent PCR test result in order to calculate an odds ratio for reinfection. We estimated an odds ratio for reinfection ranging from 0.14 (95% CI: 0.019 to 0.63) to 0.28 (95% CI: 0.05 to 1.1), implying that the presence of SARS-CoV-2 antibodies at baseline is associated with around 72% to 86% reduced odds of a subsequent PCR positive test based on our point estimates. This suggests that primary infection with SARS-CoV-2 provides protection against reinfection in the majority of individuals, at least over a 6-month time period. We also highlight 2 major sources of bias and uncertainty to be considered when estimating the relative risk of reinfection, confounders and the choice of baseline time point, and show how to account for both in reinfection analysis.

## Abstract 2

Identifying the potential for SARS-CoV-2 reinfection is crucial for understanding possible long-term epidemic dynamics. Analysis of a seroepidemiological cohort suggests that primary infection with SARS-CoV-2 protects against reinfection in the majority of individuals, at least over a six month period.

## Introduction

The rapid global spread of COVID-19 throughout 2020 occurred as a result of the introduction of a highly transmissible virus, SARS-CoV-2, into populations with little preexisting immunity [1]. Identifying the extent and duration of protective immunity afforded by natural infection is therefore of crucial importance for understanding possible long-term epidemic dynamics of SARS-CoV-2 [2].

Studies have estimated that over 95% of symptomatic COVID-19 cases develop antibodies against SARS-CoV-2, with most individuals developing antibodies within 3 weeks of symptom onset [3,4]. Several serological studies have also characterised individual-level immune dynamics, with some finding evidence for antibody waning and others for sustained antibody responses over several months [5–10]. Antibody kinetics are thought to vary between individuals and are possibly associated with severity of illness, where asymptomatic or mildly symptomatic individuals may develop lower levels of antibodies that wane more rapidly [3,7,11]. While neutralising antibodies are thought to be associated with protection from reinfection, there are still limited studies on the impact of postinfection seropositivity on future reinfection risk [12]. Confirmed cases of reinfection with SARS-CoV-2 have been reported since August 2020 [13]. However, existing large studies examining the relative risk of reinfection in antibody positive individuals have typically involved specific cohorts who may not be representative of the wider community, such as closed communities or healthcare worker cohorts [14–17]. To evaluate the relative risk of SARS-CoV-2 infection and reinfection over time, we analysed PCR and serological testing data from a prospective cohort of SpaceX employees in the US between April 2020 and February 2021 [18,19].

## Results

**Fig 1:**
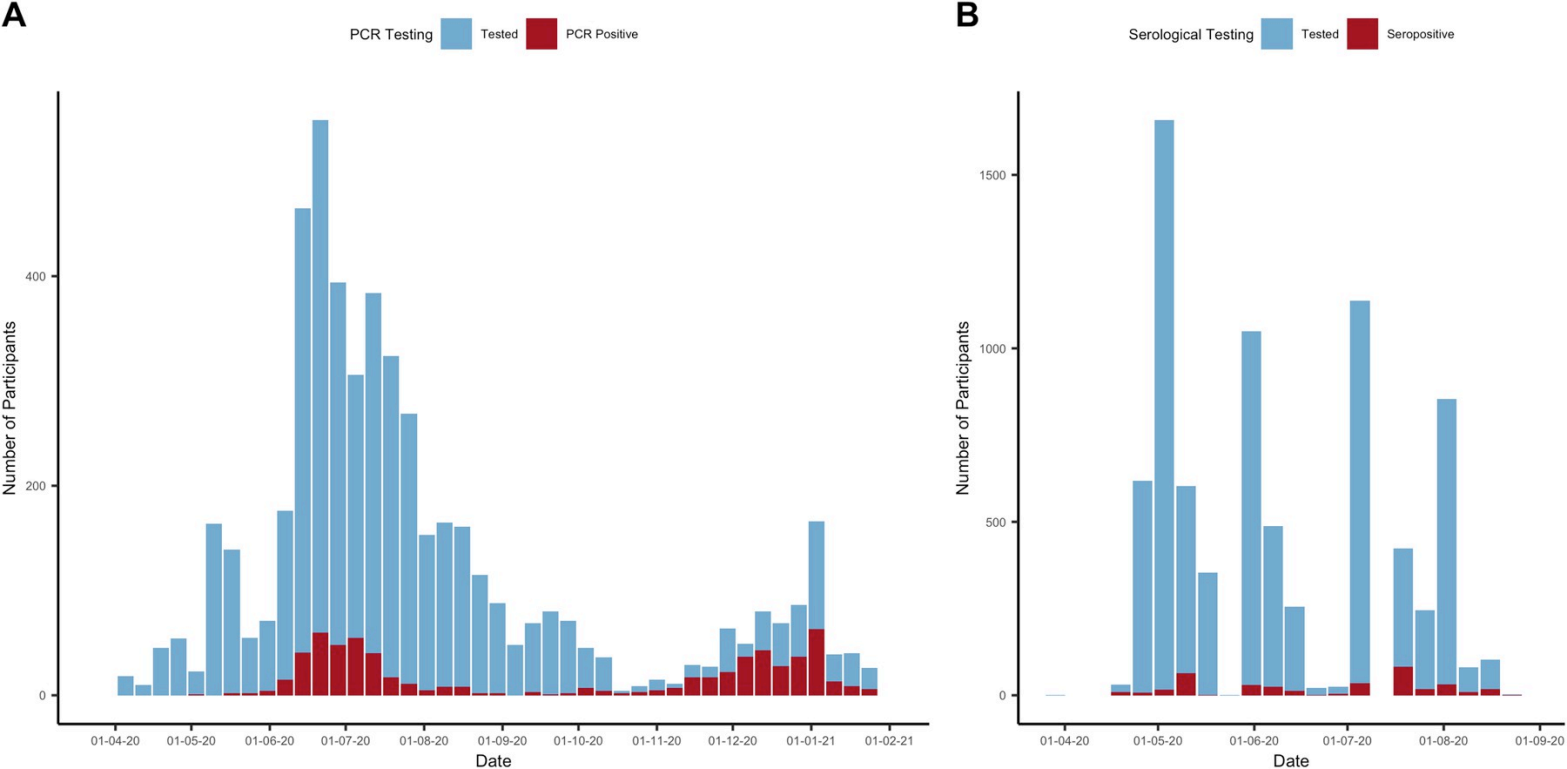
**(A)** Number of PCR tests and PCR positive tests in the cohort between April 5, 2020 and January 31, 2021 from 3,296 participants. **(B)** Number of serological tests and seropositive tests between March 29, 2020 and August 23, 2020 from 4,411 participants. Data underlying this figure can be found in https://github.com/EmilieFinch/covid-reinfection.

**Fig 2:**
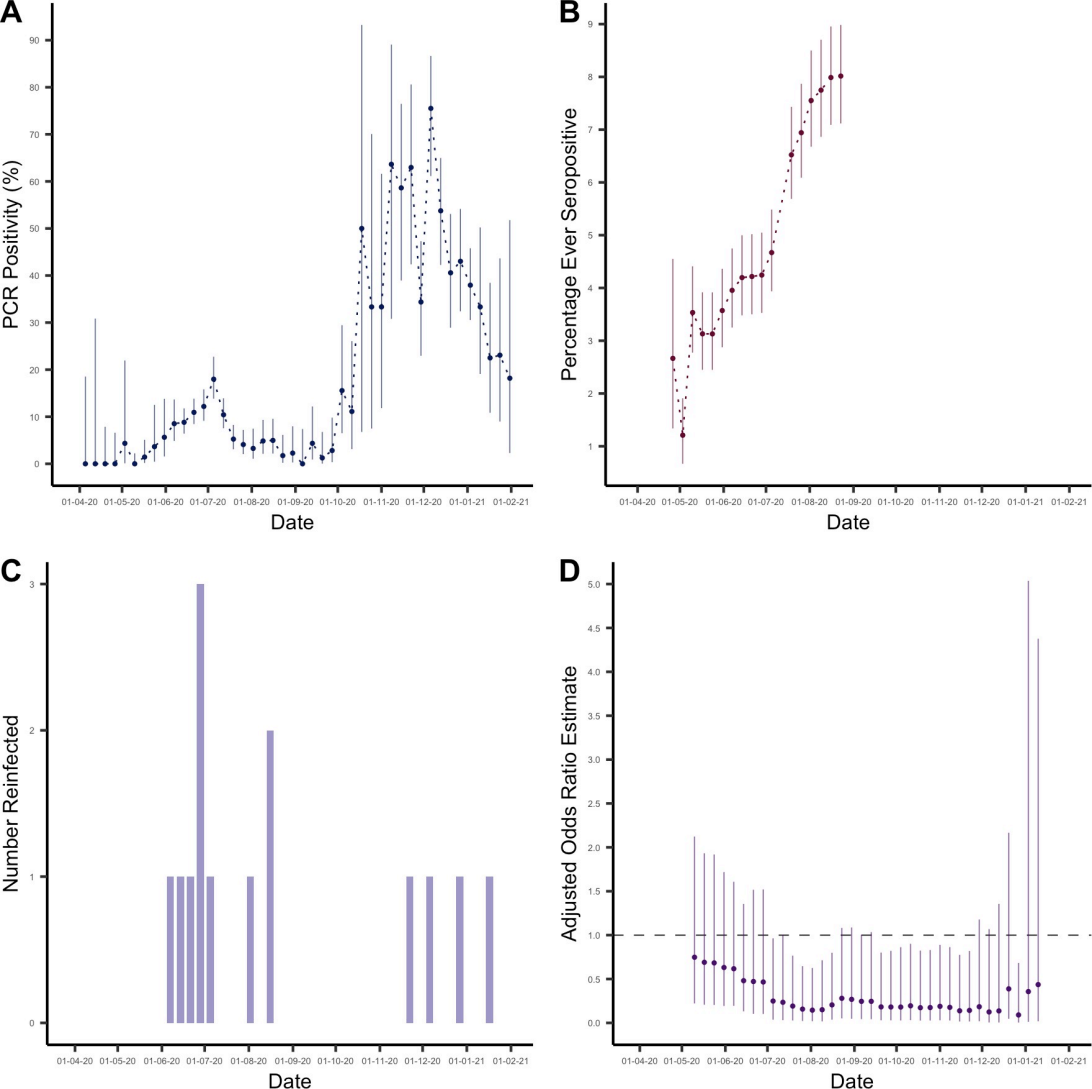
**(A)** PCR positivity (%) in the cohort between April 5, 2020 and January 31, 2021. **(B)** Percentage ever seropositive in the cohort (number ever seropositive/cumulative number enrolled) between March 29, 2020 and August 23, 2020. Note that the percentage ever positive decreases initially as participants continue to be enrolled in the study. **(C)** Number of possible reinfections in cohort over time (defined as a new positive PCR test more than 30 days after initial seropositive result). **(D)** Odds ratio estimates comparing odds of reinfection in the seropositive group with odds of primary infection in the seronegative group, estimated using logistic regression and adjusted for potential confounders. The estimates are presented with their associated 95% CIs and with the cutoff week used to define baseline seroprevalence on the x-axis. Data underlying this figure can be found in https://github.com/EmilieFinch/covid-reinfection.

Of 4,411 individuals enrolled, 309 individuals tested seropositive during the study period (Fig 1). This resulted in an overall adjusted percentage ever seropositive of 8.2% (95% CI: 7.3% to 9.1%) by the end of August 2020, after the final round of serological testing (Fig 2B). Here, imperfect test sensitivity and specificity were adjusted for using the Rogan–Gladen correction [20]. We defined a possible reinfection as a new positive PCR test more than 30 days after initial seropositive result. This identified 14 possible reinfections with a median time of 66.5 days between initial seropositive test and PCR positive test (Fig 2C).

### SARS-CoV-2 infection and reinfection

We estimated the odds ratio for SARS-CoV-2 reinfection using multivariable logistic regression, to adjust for any background individual-level variation in the risk of infection (see Methods). This required us to choose a cutoff week in order to define baseline seroprevalence and the subsequent observation period for PCR testing. To examine how our estimate for the odds ratio for reinfection varied depending on the cutoff week chosen, we repeated the analysis using every possible cutoff week.

We considered that the most robust estimation of the odds ratio for reinfection would occur midepidemic when using cutoff weeks in between 2 “waves” of the epidemic seen in the study cohort. We validated this methodological assumption by conducting a simulation study (see Fig A in S2 Text).

We defined a midepidemic period in between 2 epidemic waves where PCR positivity in the study cohort was below the WHO specified threshold of 5%, which occurred between July 26, 2020 and September 27, 2020 (Fig 2A). During these cutoff weeks, estimates of the odds ratio for reinfection (Fig 2D) ranged from 0.14 (95% CI: 0.019 to 0.63) to 0.28 (95% CI: 0.05 to 1.1). Our point estimates suggest that the presence of SARS-CoV-2 antibodies confers around 72% to 86% protection against reinfection with SARS-CoV-2, at least over a 6-month period. As a sensitivity analysis, we conducted the same analysis but excluding records where individuals had recorded a specific trigger reason for testing such as symptom onset or potential exposure (and so reflecting individuals tested at random). Considering the weeks between July 26, 2020 and September 27, 2020, we found estimates of the odds ratio for reinfection ranged from 0.18 (95% CI: 0.024 to 0.80) to 0.36 (95% CI: 0.06 to 1.5).

In the adjusted analyses, odds ratio estimates for reinfection converged to similar values for cutoffs spanning a period after the first peak of infection in early July. By this point, sufficient numbers of participants had been both recruited and tested seropositive (see Fig 2B) that we had enough data to distinguish infection dynamics in seropositive and seronegative groups. Adjusted odds ratio estimates for reinfection then lost precision when using late cutoff weeks from mid-December onwards due to increasingly small numbers of participants experiencing PCR infection after the cutoff point, consistent with our simulation study (see S2 Text).

**Fig 3:**
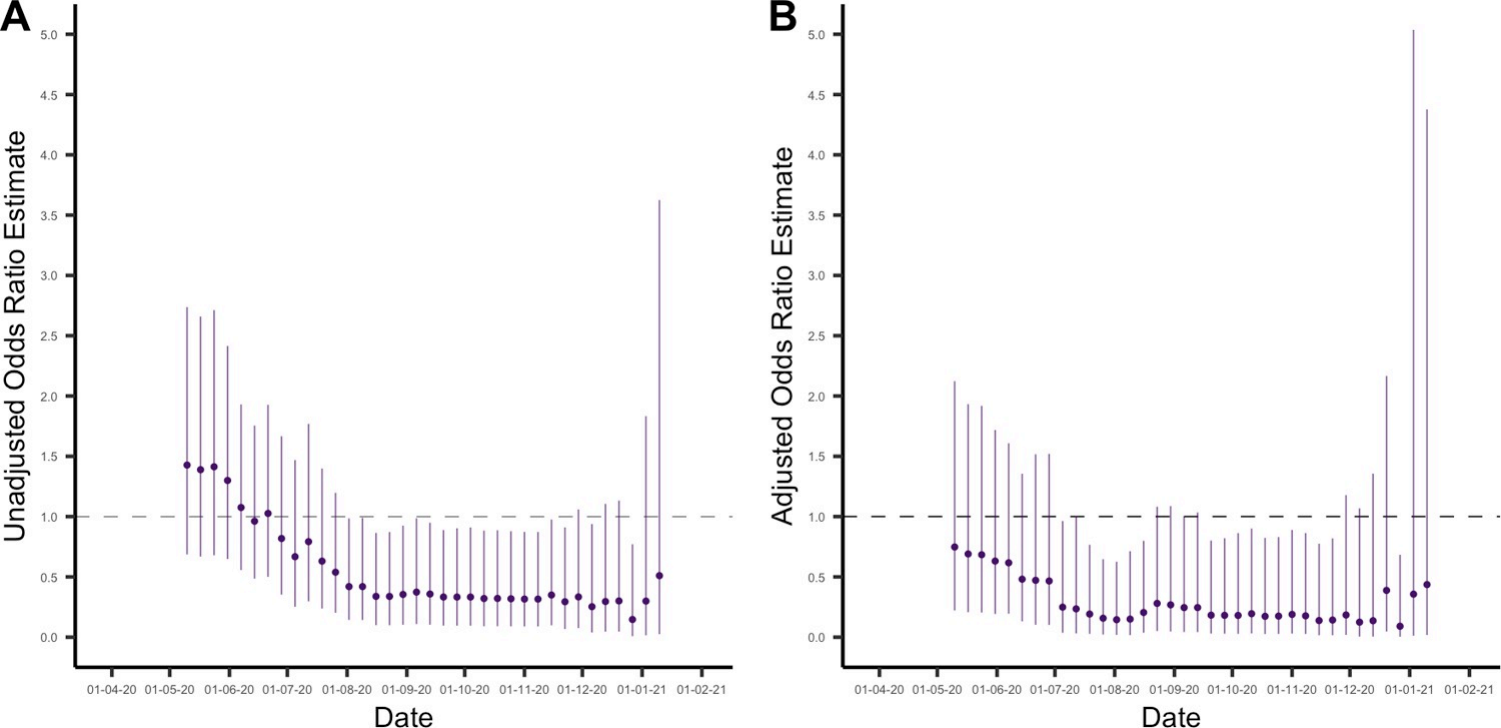
**(A)** Unadjusted odds ratio estimates comparing odds of reinfection in the seropositive group with odds of primary infection in the seronegative group. The estimates are presented with their associated 95% CIs and with the cutoff week used to define baseline seroprevalence on the x-axis. **(B)** Odds ratio estimates comparing odds of reinfection in the seropositive group with odds of primary infection in the seronegative group, estimated using logistic regression and adjusted for potential confounders. The estimates are presented with their associated 95% CIs and with the cutoff week used to define baseline seroprevalence on the x-axis. Data underlying this figure can be found in https://github.com/EmilieFinch/covid-reinfection.

Unadjusted odds ratio estimates tended to overestimate the odds ratio for reinfection compared with primary infection, particularly when using early cutoff weeks (Fig 3). Notably, with early cutoff weeks the unadjusted analysis estimated a higher odds of reinfection compared to primary infection, albeit with wide CIs. This is the result of a subset of individuals who are at higher risk of initial seroconversion (who would be included in analyses at earlier time thresholds) and also at higher risk of later reinfection, giving a biased estimate of the association between antibodies and subsequent infection when using earlier cutoff weeks.

## Discussion

We identified 14 possible reinfections out of 309 seropositive individuals in the prospective seroepidemiological cohort between April 2020 and February 2021, estimating an odds ratio for reinfection ranging from 0.14 (95% CI: 0.019 to 0.63) to 0.28 (95% CI: 0.05 to 1.1). This provides evidence that primary infection with SARS-CoV-2 results in protection against reinfection in the majority of individuals, at least over a sixth month time period. Our findings are broadly consistent with estimates of 0.17 (95% CI 0.13 to 0.24) odds ratio [14] and 0.11 (0.03 to 0.44) incidence rate ratio [15] for healthcare workers, 0.18 (0.11 to 0.28) incidence rate ratio for military recruits [16], and 0.195 (95% CI 0.155 to 0.246) incidence rate ratio from a Danish population-level study [21].

Our analysis addressed 2 key sources of bias and uncertainty in estimating the relative risk of reinfection. First, confounders may inflate estimates; if a specific subset of the cohort is at higher risk of infection (e.g., due to underlying health conditions or increased risk of exposure), these participants will be more likely to be both initially seropositive and to have a subsequent reinfection. Second, the time period considered could increase uncertainty; defining the baseline seroprevalence at an early time point means few will be seropositive, whereas defining it at a later point means there is less time to observe possible reinfections. We accounted for these 2 factors by adjusting for key confounders to calculate an adjusted odds ratio for reinfection. We then investigated how changing the cutoff date to define baseline seroprevalence impacted the accuracy of the adjusted odds ratio calculated. We assumed that for a 2-wave epidemic scenario, a cutoff week in the period in between the 2 waves of infection risk would result in the most robust estimates of the odds ratio for reinfection, which we validated using a simulation study (see S2 Text). This suggests that the robustness of estimates of the relative risk of reinfection will be sensitive to the study period chosen, relative to population-level epidemic dynamics.

There are several limitations to the underlying data that should be considered when interpreting these findings. This prospective cohort was recruited opportunistically from employees at one US company and is unlikely to be representative of the general population. However, as we did not identify any workplace outbreaks, infections in this cohort are likely to be more reflective of community transmission than in healthcare worker cohorts or other specialised populations. Additionally, we only considered possible reinfections (as opposed to probable or true reinfections). As possible reinfections did not meet a stringent case definition, such as confirmation through genomic sequencing, they may include cases of prolonged viral shedding following an initial infection. This would result in an overestimation of the odds ratio for reinfection and so our analysis reflects the minimum possible effect of antibodies on future SARS-CoV-2 infection risk. Finally, the date of infection among seropositive participants is unknown, limiting inference on exact duration of protection.

As well as quantifying the relative risk of reinfection over a 6-month period among a prospectively followed workplace population, our study highlights the importance of accounting for both individual-level heterogeneity in infection risk and population-level variation in epidemic dynamics when assessing the potential for reinfections.

## Methods

### Seroepidemiological cohort description

We used data from a seroepidemiological study of US employees at SpaceX, as described previously [19]. In brief, this study involved employees from 7 work locations in California, Florida, Texas, and Washington State, with ages ranging from 18 to 71. A total of 4,411 employees volunteered to participate in the study and were enrolled from approximately 8,400 total employees. All employees were invited to participate by email, and there were no exclusion criteria. Study participants were offered SARS-CoV-2 IgG receptor binding domain (RBD) antibody testing with an in-house ELISA assay with 82.4% sensitivity and 99.6% specificity [22]. Serological samples were taken during 4 rounds of testing between April and September 2020. A questionnaire including demographic, symptom, and exposure information was conducted at enrolment and with each round of serological testing. Individuals continued to be enrolled throughout the study period, and around half of the total participants (48%) were tested at more than 1 time point. Participants occupied a range of job positions within SpaceX including office-based and factory-based jobs. Additionally, symptomatic and asymptomatic PCR testing were widely available for employees using the Infinity BiologiX (IBX) TaqPath rRT-PCR assay, with data available from April 2020 to January 2021. Employees could request a test for any reason, and testing was also specifically performed for symptomatic individuals, individuals with potential exposure, and mission critical employees. Both serology and PCR testing data were available for 1,800 individuals.

### Ethics statement

The study protocol was approved by the Western Institutional Review Board (ref 20200991). The use of deidentified data and biological samples was approved by the Mass General Brigham Healthcare Institutional Review Board (ref 2020P001166). Secondary data analysis was approved by the LSHTM Observational Research Ethics Committee (ref 22466). All participants provided written informed consent.

### Statistical analysis

To estimate the odds ratio for SARS-CoV-2 reinfection, we conducted multivariable logistic regression analysis investigating the association between baseline serological status and subsequent PCR test result, given a test was sought.

The choice of cutoff week used to define participants’ baseline seroprevalence and the subsequent observation period for PCR testing have important implications in the estimation of the odds ratio for reinfection. For instance, a cutoff week early in the study period will result in few seropositive individuals, while a cutoff week later in the study period leaves less time to observe subsequent PCR testing and detect possible reinfections, impacting the accuracy of estimates. To assess how the choice of cutoff week affected estimates of the odds ratio for reinfection, we repeated the multivariable logistic regression for every possible cutoff week. We assumed the most robust estimation of the relative risk of reinfection would occur in the between the 2 “waves” of infection risk seen in the study cohort. We validated this assumption by conducting a simulation analysis using a known underlying probability distribution of infection and reinfection (see S2 Text).

Potential confounding variables included age, sex, race, ethnicity, BMI, state, work location, job category, household size, history of chronic disease, history of smoking, and test frequency. We used a backwards selection procedure to select which variables to adjust for in our analyses, minimising root mean square error (RMSE) at each step [23]. Age and sex were considered “forced” variables, which we decided to control for a priori and were adjusted for in all analyses [24,25]. We conducted variable selection separately for each cutoff week and the variable sets adjusted for in each regression analysis are listed in Table A in S1 Text. For most cutoff weeks (specifically those between May 19, 2020 and November 22, 2020), all potential confounders were adjusted for, while early weeks (between April 26, 2020 and May 3, 2020) and late weeks (between December 20, 2020 and January 17, 2021) adjusted for a subset of potential confounders.

As a sensitivity analysis, we performed the same analysis but excluding records where individuals had recorded a specific reason for test such as onset of symptoms or potential exposure to a COVID-19 case. As such, this sensitivity analysis included only individuals tested at random.

**Fig 4:**
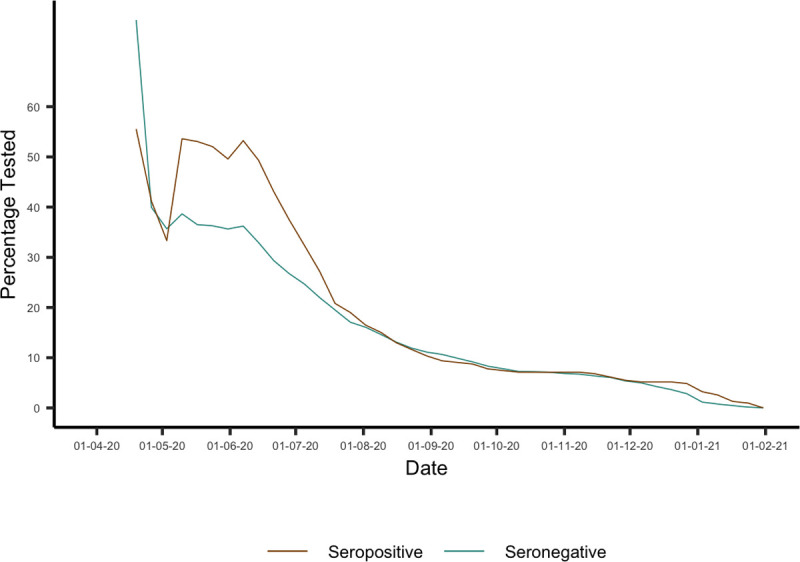
Propensity to seek a PCR test between the seronegative and seropositive groups, for each cutoff week considered in the main analysis. This was calculated as the percentage of those enrolled by the cutoff week shown on the x-axis who received at least 1 PCR test in the subsequent observation period. Data underlying this figure can be found in https://github.com/EmilieFinch/covid-reinfection.

We investigated the propensity to be tested among seronegative and seropositive individuals for each cutoff week by examining the percentage of those enrolled in the study by each cutoff week who had at least 1 test in the subsequent observation period and found that for cutoff weeks from mid-July onwards they were broadly similar between the 2 groups (Fig 4). However, the average distribution of test frequency differed between the seropositive and seronegative groups, with higher frequency of testing more common in the seronegative group. To account for this, we included PCR test frequency as a potential confounder in our analysis, defined as the number of PCR tests each individual took during the observation period (1 to 2, 3 to 5, or 6+). Protection against infection with SARS-CoV-2 conferred by the presence of antibodies was estimated such that

$${Protection}_{AB}=1-{AOR}_{Reinfection}$$

Analysis was conducted in R version 4.0.3. Code to reproduce the figures and simulation analysis presented here can be found at https://github.com/EmilieFinch/covid-reinfection.

## Supporting information

S1 Text Confounder adjustment for logistic regression analyses. (DOCX)

S2 Text Description of simulation analysis investigating how the estimation of the relative risk of reinfection varies depending on population-level epidemic dynamics. (DOCX)

## Abbreviations

COVID-19: Coronavirus Disease 2019
IBX: Infinity BiologiX
RBD: receptor binding domain
RMSE: root mean square error
SARS-CoV-2: Severe Acute Respiratory Syndrome Coronavirus 2
