# Supplementary material for: SARS-CoV-2 antibodies protect against reinfection for at least 6 months in a multicentre seroepidemiological workplace cohort
Source: PLoS Biol. 2022 Feb 10;20(2):e3001531. doi: 10.1371/journal.pbio.3001531 (PMC8865659; doi:10.1371/journal.pbio.3001531)
Supplement: S1 Text — (DOCX) [file pbio.3001531.s001.docx]

**S1 Text: Confounder adjustment for logistic regression analyses**

**Table A: Confounding variables adjusted for in logistic regression analysis for each cut-off week.**

Variable selection was conducted separately for each cut-off week. The final variable set adjusted for was selected using backwards selection minimising RMSE at each step from a list of potential confounders selected a priori. These included: age, sex, race, ethnicity, BMI, state, work location, job category, household size, history of chronic disease, history of smoking and test frequency. Age and sex were considered ‘forced’ variables and were adjusted for in all analyses.

| Date | Variables adjusted for |
| --- | --- |
| 26/04/2020 | Sex, age, BMI, history of chronic disease, household size, job category |
| 03/05/2020 | Sex, age, history of chronic disease, household size, job category, race, history of smoking |
| 10/05/2020 | Sex, age, state, BMI, race, ethnicity, job category, test frequency, history of chronic disease, household size, smoking |
| 17/05/2020 | Sex, age, state, BMI, race, ethnicity, job category, test frequency, history of chronic disease, household size, smoking |
| 24/05/2020 | Sex, age, state, BMI, race, ethnicity, job category, test frequency, history of chronic disease, household size, smoking |
| 31/05/2020 | Sex, age, state, BMI, race, ethnicity, job category, test frequency, history of chronic disease, household size, smoking |
| 07/06/2020 | Sex, age, state, BMI, race, ethnicity, job category, test frequency, history of chronic disease, household size, smoking |
| 14/06/2020 | Sex, age, state, BMI, race, ethnicity, job category, test frequency, history of chronic disease, household size, smoking |
| 21/06/2020 | Sex, age, state, BMI, race, ethnicity, job category, test frequency, history of chronic disease, household size, smoking |
| 28/06/2020 | Sex, age, state, BMI, race, ethnicity, job category, test frequency, history of chronic disease, household size, smoking |
| 05/07/2020 | Sex, age, state, BMI, race, ethnicity, job category, test frequency, history of chronic disease, household size, smoking |
| 12/07/2020 | Sex, age, state, BMI, race, ethnicity, job category, test frequency, history of chronic disease, household size, smoking |
| 19/07/2020 | Sex, age, state, BMI, race, ethnicity, job category, test frequency, history of chronic disease, household size, smoking |
| 26/07/2020 | Sex, age, state, BMI, race, ethnicity, job category, test frequency, history of chronic disease, household size, smoking |
| 02/08/2020 | Sex, age, state, BMI, race, ethnicity, job category, test frequency, history of chronic disease, household size, smoking |
| 09/08/2020 | Sex, age, state, BMI, race, ethnicity, job category, test frequency, history of chronic disease, household size, smoking |
| 16/08/2020 | Sex, age, state, BMI, race, ethnicity, job category, test frequency, history of chronic disease, household size, smoking |
| 23/08/2020 | Sex, age, state, BMI, race, ethnicity, job category, test frequency, history of chronic disease, household size, smoking |
| 30/08/2020 | Sex, age, state, BMI, race, ethnicity, job category, test frequency, history of chronic disease, household size, smoking |
| 06/09/2020 | Sex, age, state, BMI, race, ethnicity, job category, test frequency, history of chronic disease, household size, smoking |
| 13/09/2020 | Sex, age, state, BMI, race, ethnicity, job category, test frequency, history of chronic disease, household size, smoking |
| 20/09/2020 | Sex, age, state, BMI, race, ethnicity, job category, test frequency, history of chronic disease, household size, smoking |
| 27/09/2020 | Sex, age, state, BMI, race, ethnicity, job category, test frequency, history of chronic disease, household size, smoking |
| 04/10/2020 | Sex, age, state, BMI, race, ethnicity, job category, test frequency, history of chronic disease, household size, smoking |
| 11/10/2020 | Sex, age, state, BMI, race, ethnicity, job category, test frequency, history of chronic disease, household size, smoking |
| 18/10/2020 | Sex, age, state, BMI, race, ethnicity, job category, test frequency, history of chronic disease, household size, smoking |
| 25/10/2020 | Sex, age, state, BMI, race, ethnicity, job category, test frequency, history of chronic disease, household size, smoking |
| 01/11/2020 | Sex, age, state, BMI, race, ethnicity, job category, test frequency, history of chronic disease, household size, smoking |
| 08/11/2020 | Sex, age, state, BMI, race, ethnicity, job category, test frequency, history of chronic disease, household size, smoking |
| 15/11/2020 | Sex, age, state, BMI, race, ethnicity, job category, test frequency, history of chronic disease, household size, smoking |
| 22/11/2020 | Sex, age, state, BMI, race, ethnicity, job category, test frequency, history of chronic disease, household size, smoking |
| 29/11/2020 | Sex, age, state, BMI, race, ethnicity, job category, test frequency, history of chronic disease, household size, smoking |
| 06/12/2020 | Sex, age, state, BMI, race, ethnicity, job category, test frequency, history of chronic disease, household size, smoking |
| 13/12/2020 | Sex, age, state, BMI, race, ethnicity, job category, test frequency, history of chronic disease, household size, smoking |
| 20/12/2020 | Sex, age, BMI, history of chronic disease, ethnicity, household size, job category, history of smoking, state, test frequency |
| 27/12/2020 | Sex, age, BMI, history of chronic disease, ethnicity, household size, smoking, state, test frequency |
| 03/01/2021 | Sex, age, ethnicity, household size, smoking |
| 10/01/2021 | Sex, age, BMI, state, test frequency |
| 17/01/2021 | Sex, age, BMI, race, state |

**
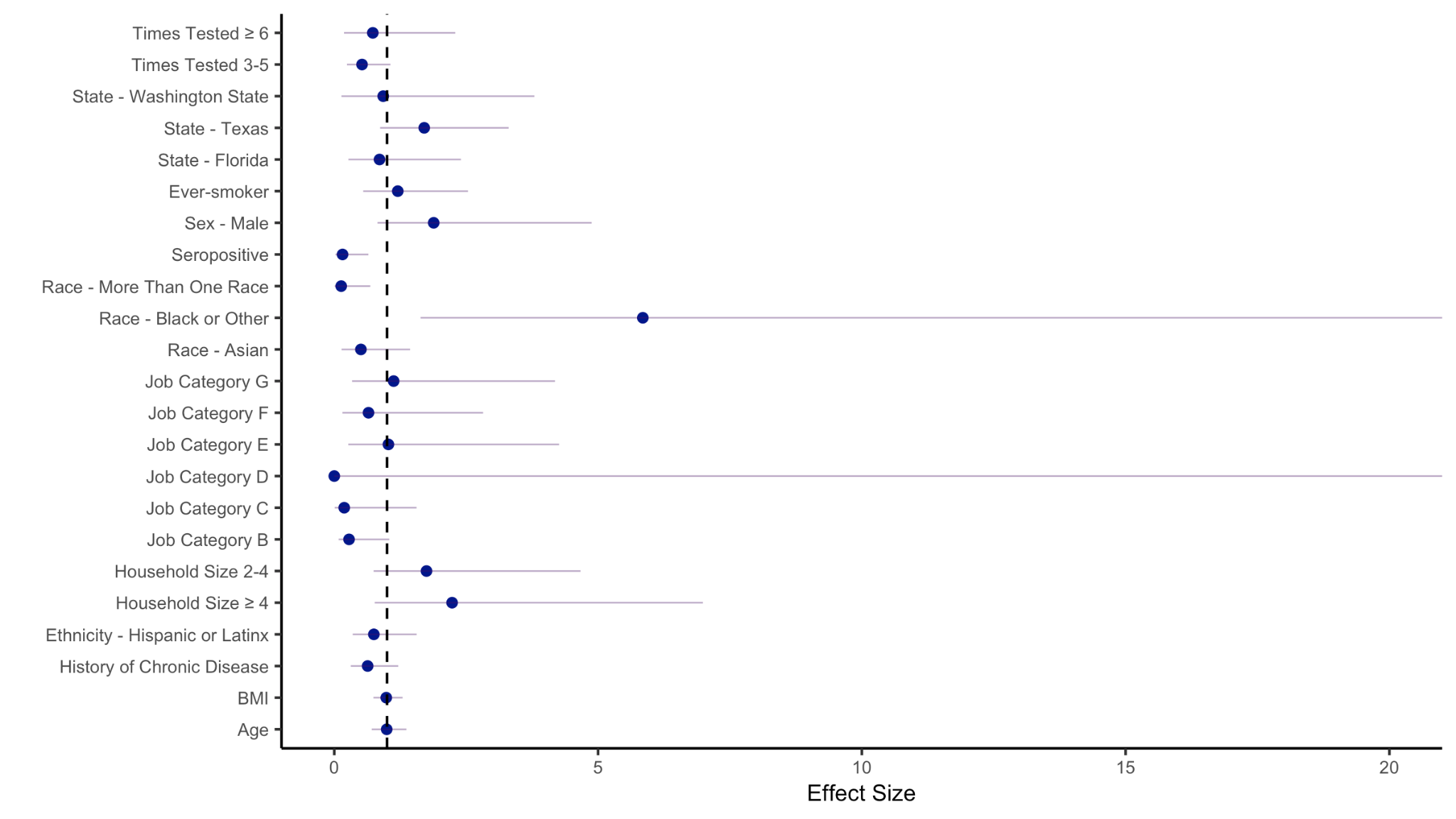
**

**Figure A: Effect sizes of confounders adjusted for in main analysis for the cut-off week of 26/7/2021.** Estimates are presented with their associated 95% confidence intervals. The upper bound of the 95% confidence interval for ‘Race - Black or Other’ of 23.7 is not shown. The estimate of ‘Job Category D’ is not well defined due to a low sample size, and the upper bound is not shown. Reference categories for categorical variables are: Times Tested 1-2; State - California; Never-smoker; Sex - Female; Seronegative; Race - White; Job Category A; Household Size 1; Ethnicity - Not Hispanic/Not Latinx; No History of Chronic Disease. Job categories have been anonymised for data protection.


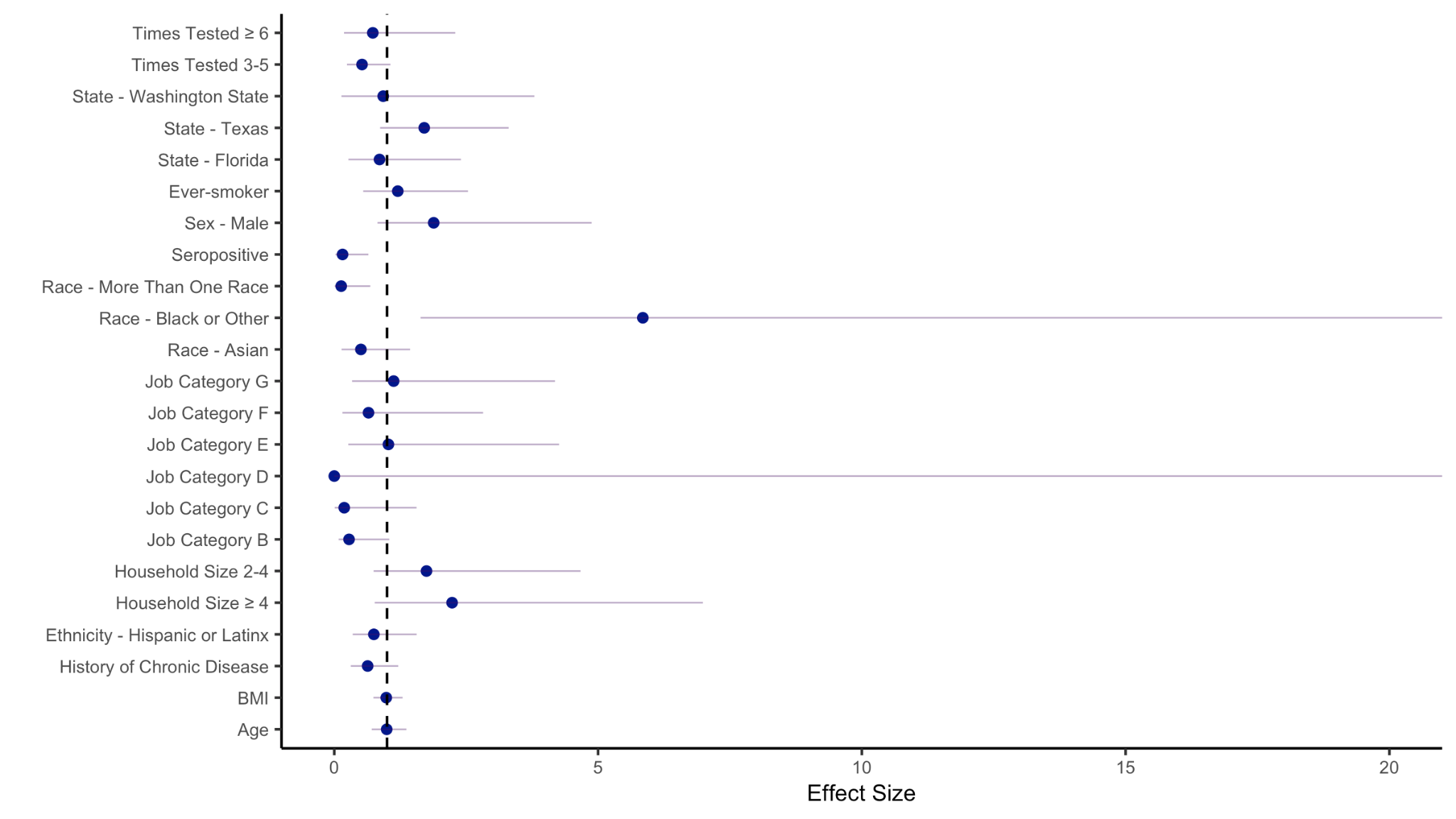


**Figure B: Effect sizes of confounders adjusted for in main analysis for the cut-off week of 16/8/2020.** Estimates are presented with their associated 95% confidence intervals. The upper bound of the 95% confidence interval for ‘Race - Black or Other’ of 51.4 is not shown. The estimate of ‘Job Category D’ is not well defined due to a low sample size, and the upper bound is not shown. Reference categories not shown for categorical variables are: Times Tested 1-2; State - California; Never-smoker; Sex - Female; Seronegative; Race - White; Job Category A; Household Size 1; Ethnicity - Not Hispanic/Not Latinx; No History of Chronic Disease. Job categories have been anonymised for data protection.


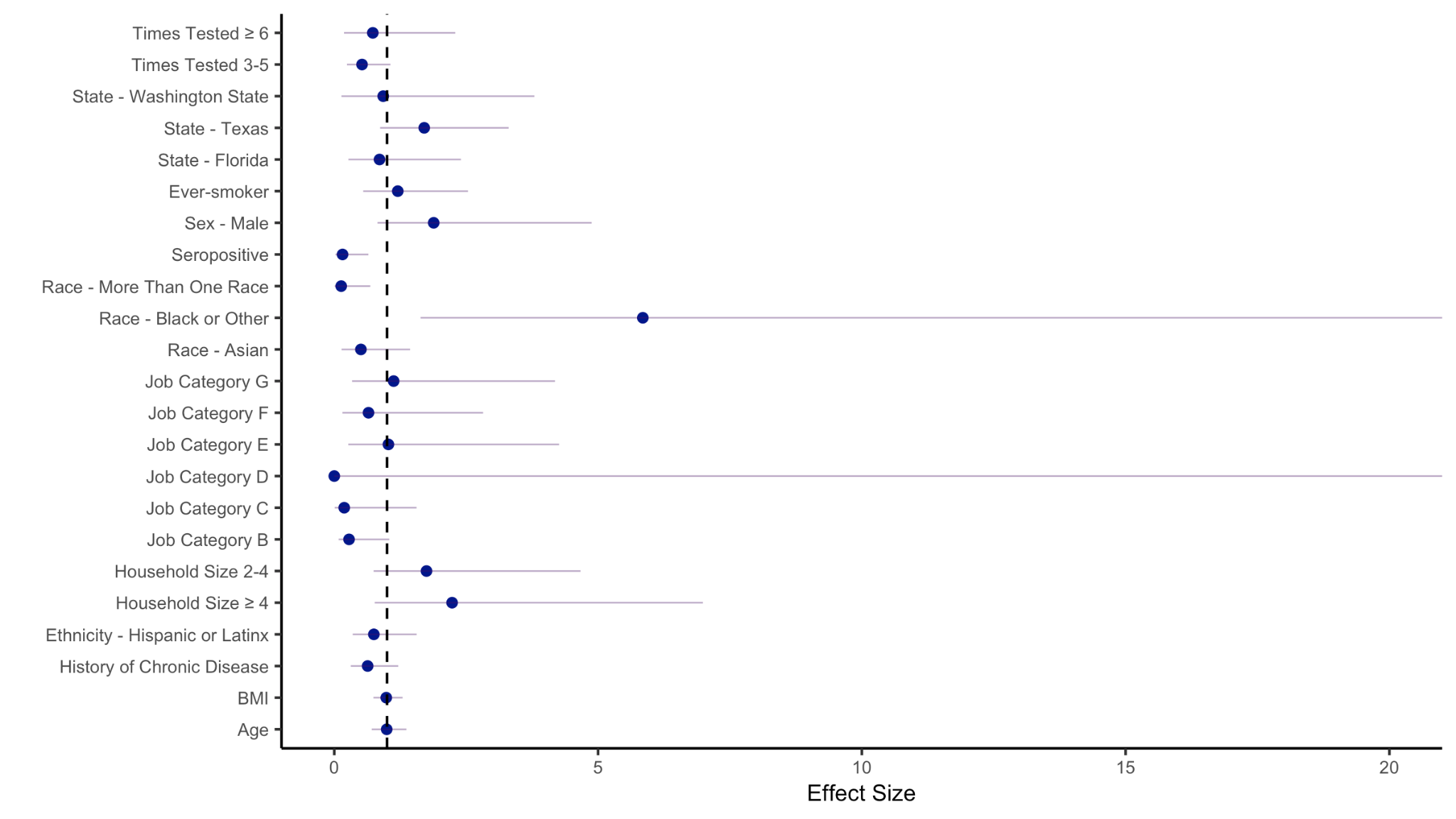


**Figure C: Effect sizes of confounders adjusted for in main analysis for the cut-off week of 13/9/2020.** Estimates are presented with their associated 95% confidence intervals. The upper bound of the 95% confidence interval for ‘Race - Black or Other’ of 93.7 is not shown. The estimate of ‘Job Category D’ is not well defined due to a low sample size, and the upper bound is not shown. Reference categories not shown for categorical variables are: Times Tested 1-2; State - California; Never-smoker; Sex - Female; Seronegative; Race - White; Job Category A; Household Size 1; Ethnicity - Not Hispanic/Not Latinx; No History of Chronic Disease. Job categories have been anonymised for data protection.
