# Supplementary material for: SARS-CoV-2 antibodies protect against reinfection for at least 6 months in a multicentre seroepidemiological workplace cohort
Source: PLoS Biol. 2022 Feb 10;20(2):e3001531. doi: 10.1371/journal.pbio.3001531 (PMC8865659; doi:10.1371/journal.pbio.3001531)
Supplement: S2 Text — (DOCX) [file pbio.3001531.s002.docx]

**S2 Text: Description of simulation analysis investigating how the estimation of the relative risk of reinfection varies depending on population-level epidemic dynamics**

We conducted a simulation analysis to validate our methodological assumption that the most robust estimation of the odds ratio for reinfection would occur in the mid-epidemic period, and to

further investigate how the estimation of the odds ratios for reinfection varies depending on population-level epidemic dynamics.

We simulated a two-wave epidemic scenario using a cumulative probability distribution for infection derived from PCR testing data from the study cohort (see Figure 2A in the main text). This was scaled so the overall cumulative probability of infection reflected the overall percentage ever-seropositive in the cohort (8%). We considered a sample size of 2000 individuals over a period of 44 weeks. For each time point *i*, the number of seropositive and seronegative individuals up to and including that time point was simulated, according to the cumulative probability of infection at time point *i* (see Eq. 1-2). We then simulated how many of these seropositive and seronegative individuals would test PCR positive or negative after time point *i*, with a pre-set risk ratio for reinfection of 0.15 (see Eq. 3-6).

| $P\left( Seropositive \right)=\sum_{t=1}^{i} Pr(X=x_{t})$ | (1) |
| --- | --- |
| ${P(Seronegative)}= 1-\sum_{t=1}^{i} Pr(X=x_{t})$ | (2) |
| ${P(PCR Positive \vert Seronegative)}=1-\Pi(1-\sum_{t=i+1}^{n} Pr(X=x_{t}))$ | (3) |
| ${P(PCR Negative \vert Seronegative)}=1-(1-\Pi(1-\sum_{t=i+1}^{n} Pr(X=x_{t})))$ | (4) |
| ${P(PCR Positive \vert Seropositive)}=0.15 \times(1-\Pi(1-\sum_{t=i+1}^{n} Pr(X=x_{t})))$ | (5) |
| $P\left( PCR Negative \right\vert Seropositive)=1-(0.15 \times(1-\Pi(1-\sum_{t=i+1}^{n} Pr(X=x_{t}))))$ | (6) |

Finally, we re-estimated the risk ratio for reinfection for each cut-off time point and assessed the accuracy by comparing the estimated risk ratio using each cut-off week with the ‘true’ ratio of 0.15.

When considering this two-wave epidemic scenario, we found that the accuracy in the estimated risk ratio for reinfection was maximised in the middle of the simulation period (i.e. between the two ‘waves’ of infection risk). This supports our methodological assumption that the most robust estimation of the relative risk of reinfection would occur in between two ‘waves’ of infection in our study cohort, where we considered odds ratios given the need for an underlying regression analysis.

***
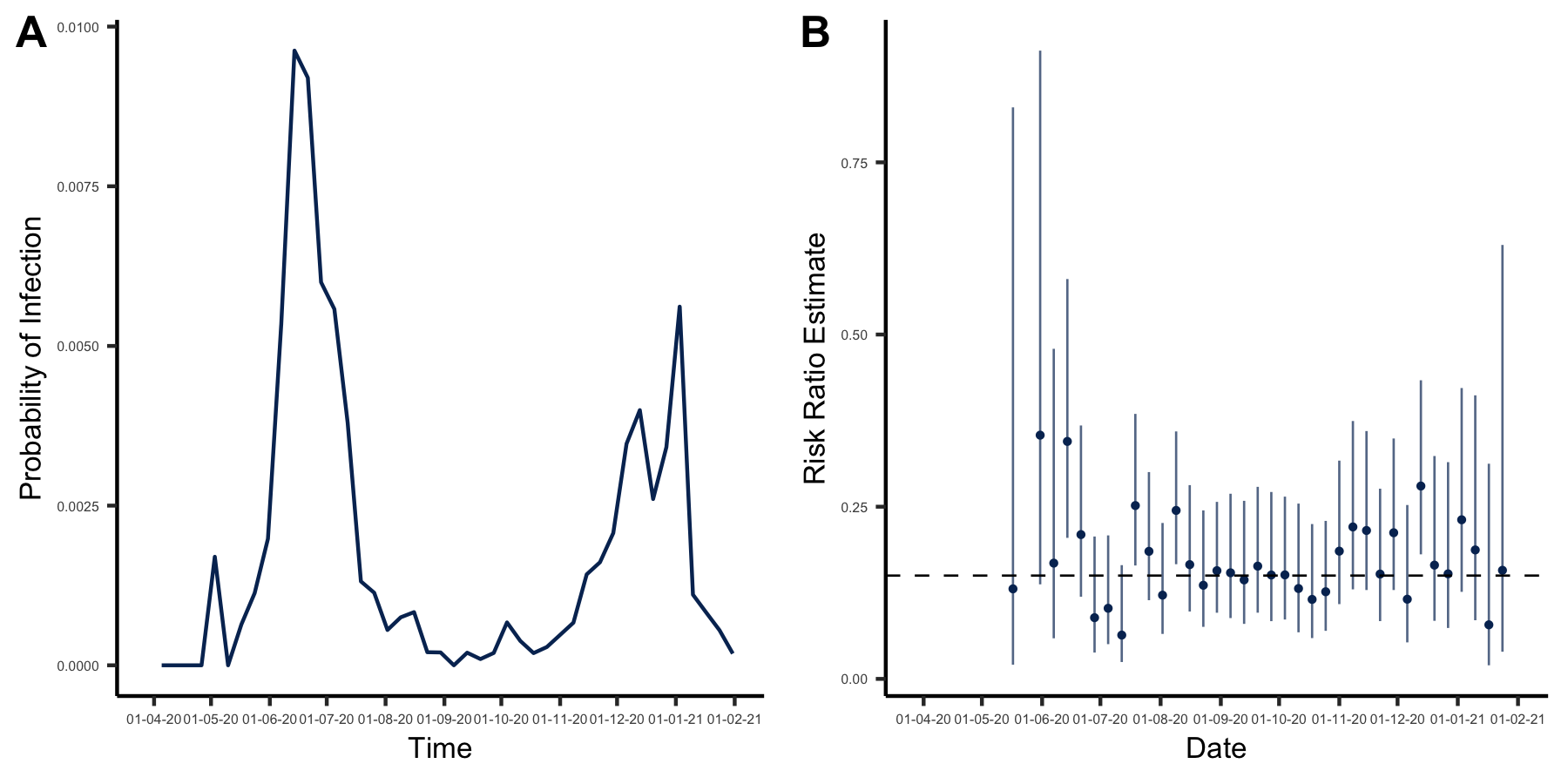
***

**Figure A: A) Probability of infection used for simulation analysis, derived from PCR testing data in the study cohort. B) Risk ratio estimates comparing the risk of reinfection with the risk of primary infection.** The estimates are presented with their associated 95% confidence intervals and with the cut-off week used to define baseline infection status on the x-axis. The dashed line represents the pre-set reinfection risk ratio of 0.15.
